# Heart disease in pregnancy and adverse outcomes: an umbrella review

**DOI:** 10.3389/fmed.2025.1489991

**Published:** 2025-02-05

**Authors:** Jiani Zhang, Yuxin Ren, Bingjie Li, Qi Cao, Xiaodong Wang, Haiyan Yu

**Affiliations:** ^1^Department of Obstetrics and Gynecology, West China Second University Hospital, Sichuan University, Chengdu, Sichuan, China; ^2^Key Laboratory of Birth Defects and Related Diseases of Women and Children (Sichuan University), Ministry of Education, Chengdu, Sichuan, China; ^3^Clinical Trial Center, National Medical Products Administration Key Laboratory for Clinical Research and Evaluation of Innovative Drugs, West China Hospital, Sichuan University, Chengdu, Sichuan, China; ^4^Department of Reproductive Medical Center, West China Second University Hospital, Sichuan University, Chengdu, Sichuan, China

**Keywords:** adverse outcomes, heart disease, meta-analysis, pregnancy, umbrella adverse outcomes, umbrella review

## Abstract

**Background:**

Heart disease in pregnancy encompasses both congenital heart disease and maternal-acquired heart disease, both of which are associated with an increased risk of various adverse outcomes for mothers and their offspring.

**Objective:**

The objective of the study was to review and summarize the evidence regarding the association between heart disease in pregnancy and adverse outcomes in mothers and their offspring.

**Data sources:**

A comprehensive search was conducted in Embase, PubMed, Web of Science, and the Cochrane Database of Systematic Reviews from inception to March 2024. The protocol for this review was registered in PROSPERO (CRD42024519144).

**Study eligibility criteria:**

This review included systematic reviews and meta-analyses that examined the association between heart disease in pregnancy and adverse outcomes for mothers and their offspring.

**Study appraisal and synthesis methods:**

Data were independently extracted by two reviewers. The quality of the systematic reviews and meta-analyses was assessed using the A Measurement Tool to Assess Systematic Reviews 2 (AMSTAR2), while Grading of Recommendations, Assessment, Development, and Evaluation (GRADE) was used to evaluate the strength of the evidence for each outcome.

**Results:**

A total of 12 meta-analyses and systematic reviews were included, which documented 156 adverse outcomes for mothers and 65 adverse outcomes for offspring. Evidence was found for both primary and secondary adverse outcomes. Adverse outcomes for mothers were death, cardiac events (cardiac arrest, heart failure, surgery, arrhythmia, anesthesia or sedation, endocarditis, mitral regurgitation, myocardial infarction, NYHA III–IV, restenosis, syncope, and others), pulmonary events (respiratory failure, pulmonary edema, and respiratory support), embolism, cerebrovascular events, postpartum hemorrhage, arterial events, delivery mode, and hospital stay. Adverse outcomes for offspring were death, pregnancy loss, growth restriction, low birth weight, preterm birth, recurrence, and uncertainty. No publication bias was detected using Egger’s test. The overall AMSTAR 2 confidence rating for the included meta-analyses and systematic reviews was moderate. The majority (55.3%) of the evidence evaluated by GRADE was of low quality, while the remaining outcomes were categorized as having “very low”-quality evidence.

**Conclusion:**

Current evidence links heart disease during pregnancy to adverse maternal outcomes, including death and cardiac, pulmonary, and cerebrovascular events, as well as increased mortality risk for offspring. Many meta-analyses in this field have limitations that raise concerns about their validity, highlighting the need for high-quality prospective studies.

## Introduction

Heart disease in pregnancy encompasses both congenital heart disease coexisting with pregnancy and maternal-acquired heart disease, which has become the most frequent cause of death during pregnancy and postpartum, outnumbering by far obstetric causes of death such as bleeding or thromboembolism (1, 2). It has been reported that maternal mortality of women with heart disease in pregnancy is much higher than that of women without such conditions (3). With advances in medical and surgical care, an increasing number of women with congenital heart disease are reaching childbearing age and considering pregnancy (4). In addition, there has been a rise in perinatal and postpartum cardiovascular complications in recent years, likely due to average maternal ages and higher prevalences of obesity and hypertension (5, 6).

Physiological adaptations in the cardiovascular system of pregnant women occur during pregnancy and childbirth, mainly manifesting in increased cardiac output, elevated circulating blood volume, and reduced peripheral vascular resistance (7). The increased cardiac load leads to an increase in new cardiovascular diseases during pregnancy or the aggravation of existing heart diseases (2). The hemodynamic changes associated with pregnancy may adversely affect both maternal and fetal/neonatal health (8). Severe clinical symptoms, such as acute coronary syndrome or aortic dissection, are characterized by the acute onset of heart disease in pregnancy, which greatly threatens the life and health of expectant mothers (9). Due to acute heart attack during pregnancy or intolerance of continuation of pregnancy, early cesarean section is often performed for timely termination of pregnancy, which triggers iatrogenic preterm labor, increasing the rate of neonatal preterm birth and associated complications. It has been declared that pregnant women with various heart diseases face higher risks of low Apgar scores, preterm labor, stillbirth, and delivering small for gestational age (SGA) infants compared to women with normal pregnancies (10, 11).

Information on the risks associated with adverse outcomes for mothers with heart disease in pregnancy, as well as the risks to their offspring, is essential for enabling both clinicians and mothers to make well-informed decisions about pregnancy management. Although there are existing studies on maternal and fetal/neonatal outcomes in the case of heart disease in pregnancy, comprehensive and precise risk assessments are still lacking.

### Objective

An umbrella review is a comprehensive method used to systematically collect, integrate, and evaluate data from multiple meta-analyses, offering a broad and nuanced perspective on the existing evidence across various health outcomes (12). To the best of our knowledge, no previous systematic review has specifically assessed the robustness, validity, or limitations of the evidence regarding adverse outcomes for women with heart disease during pregnancy and their offspring. This gap in the literature underscores the need for a more rigorous evaluation of the existing studies, particularly in terms of study quality, methodological consistency, and the potential biases inherent in previous findings. Consequently, we conducted this umbrella review to provide a thorough analysis and address the gaps in understanding the full scope of risks associated with maternal heart disease.

## Methods

### Protocol

The umbrella review was performed following the Preferred Reporting Items for Systematic Reviews and Meta-analyses (PRISMA 2020 statement) checklist (13), with the protocol registered on PROSPERO, CRD42024519144.

### Information sources and search strategy

In March 2024, four databases were systematically searched from inception: Embase, PubMed, Web of Science, and the Cochrane Database of Systematic Reviews. The search strategy used the following terms/keywords: (heart disease or cardiac disease or cardiac disorder or heart disorder) AND (pregnancy or pregnancies or gestation) AND (systematic review or meta-analysis), following the same standardized methods as seen in previously published umbrella reviews (14, 15). The reference lists of all identified articles were also manually screened.

### Eligibility criteria

We included systematic reviews and meta-analyses of women with heart disease during pregnancy and their offspring of any age or ethnicity, from any country or setting. Systematic reviews and meta-analyses of either randomized controlled trials (RCTs) or observational studies (cohort studies, case–control studies, nest case–control studies, and cross-sectional studies) were included. Meta-analyses were eligible for inclusion when they compared the effects of different cardiac diseases on the same health outcome through odds ratios, morbidity, incidence, or standardized mean differences. We included meta-analyses in which the exposure was mitral stenosis, aortic stenosis, cardiomyopathy, cardiac surgery during pregnancy, percutaneous balloon mitral valvotomy and so on. We extracted data on individual outcomes separately if two or more health outcomes or clinical settings were reported in a study. If more than one study was conducted on the same cardiac disease exposure and health outcomes, we included the most recent study for data extraction, which generally has the largest sample size, the most eligible studies, and the largest effect size.

The exclusion criteria for these umbrella reviews included systematic reviews without meta-analysis, studies with insufficient data to evaluate heart disease in pregnancy, network meta-analyses, conference abstracts, non-English studies, and animal and vitro studies.

### Data extraction

Two independent investigators (Jiani Zhang and Bingjie Li) systematically screened the titles and abstracts of the identified studies before proceeding with a full-text review to assess eligibility. Any discrepancies between the two reviewers were resolved through a discussion with a third reviewer (Qi Cao) to ensure consistency and accuracy in the selection process. Based on the lethality, severity, and medical costs associated with adverse outcomes, we categorize them into primary and secondary adverse outcomes. The following data were extracted from each eligible study: (1) primary adverse outcomes for mothers: death, cardiac events (cardiac arrest, heart failure, and surgery), pulmonary events (respiratory failure and pulmonary edema), embolism, cerebrovascular events, and postpartum hemorrhage and primary adverse outcomes for offspring: death and pregnancy loss; (2) secondary adverse outcomes for mothers: cardiac events (arrhythmia, anesthesia or sedation, endocarditis, mitral regurgitation, myocardial infarction, NYHA III–IV classification, restenosis, syncope, and other related events), pulmonary events (respiratory support), arterial events, delivery mode, and hospital stay and the secondary adverse outcomes for offspring: growth restriction, low birth weight, preterm birth, recurrence, and uncertainity; (3) type of heart disease, (4) first author’s last name; (5) publication year; (6) number of studies included in each meta-analysis; (7) number of cases or total participants included; (8) study design (i.e., cohort, case–control, randomized controlled trial [RCTs]); (9) outcome comparisons (i.e., heart disease vs. healthy controls); (10) meta-analysis metric; (11) estimated summary effect (i.e., odds ratio [OR], relative risk [RR], mean difference [MD], and prevalence), with 95% confidence interval (CI); (12) type of effect model; (13) heterogeneity; and (14) publication bias.

### Quality of systematic review and strength of evidence

The methodological quality of each included meta-analysis was evaluated using the A Measurement Tool to Assess Systematic Reviews 2 (AMSTAR 2), which classifies the quality of evidence into four categories: “high,” “moderate,” “low,” or “critically low” quality (16). We applied the Grading of Recommendations, Assessment, Development, and Evaluation (GRADE) system to assess the quality of evidence for each outcome. This system similarly categorizes evidence into “high”-, “moderate”-, “low”-, or “very low”-quality evidence (17).

### Data synthesis and assessment of risk of bias

For each meta-analysis, we reported the most adjusted estimated summary effect size with 95% CI, using either random or fixed effects models, depending on data characteristics. To assess publication bias, we used Egger’s regression asymmetry test (18). Heterogeneity among the studies was evaluated using the *I^2^* metric and Cochran’s Q-test. Given the limited statistical power of some analyses, a significance threshold of *p* < 0.10 was used for testing heterogeneity and publication bias, while a more conventional threshold of *p* < 0.05 was applied to all other statistical tests.

## Results

### Study selection

**Figure 1:**
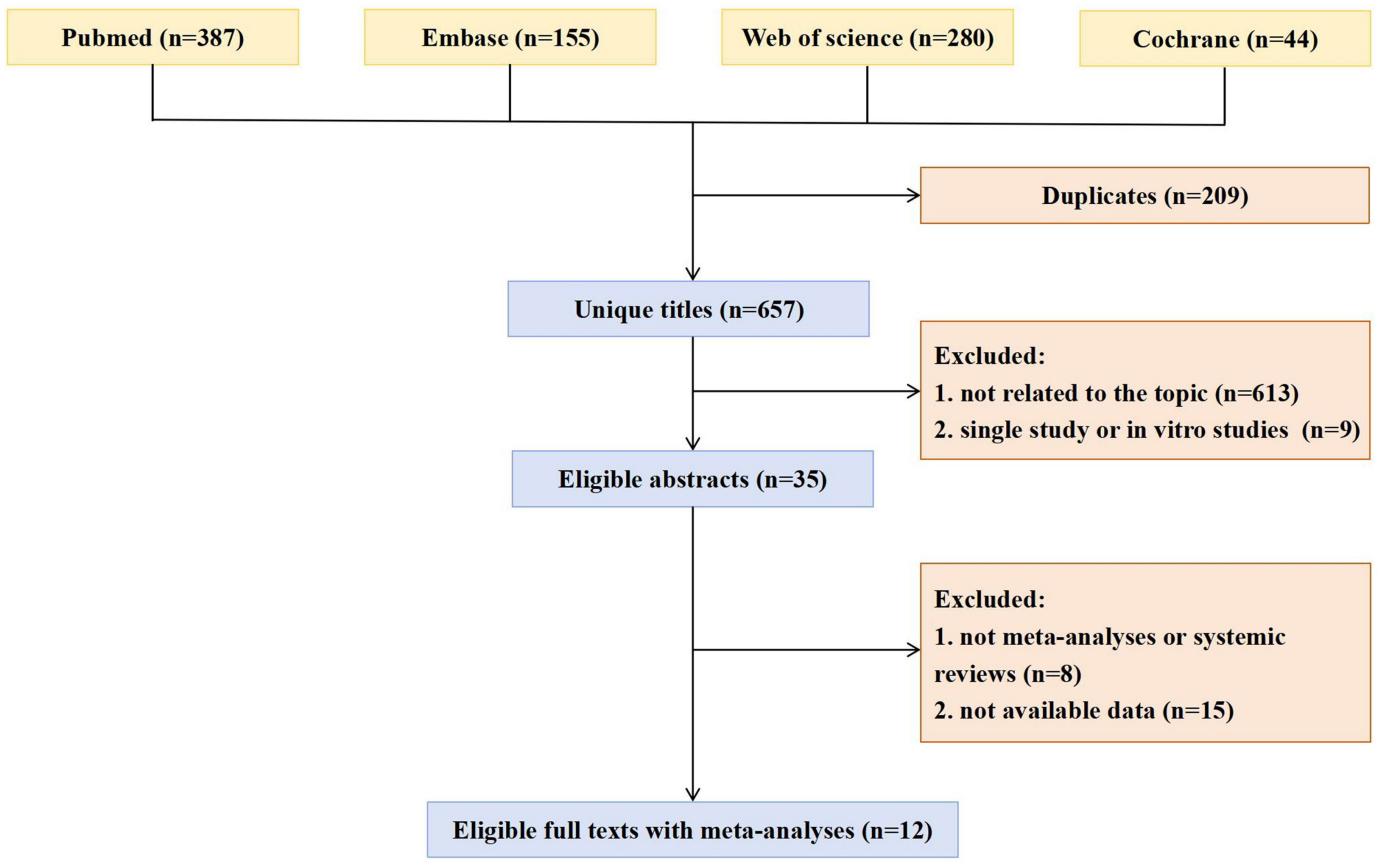
Flowchart of the selection process.

The selection process for included studies is illustrated in Figure 1. Following a systematic search and the application of inclusion and exclusion criteria, a total of 866 articles were identified and screened.

### Study characteristics

**Figure 2:**
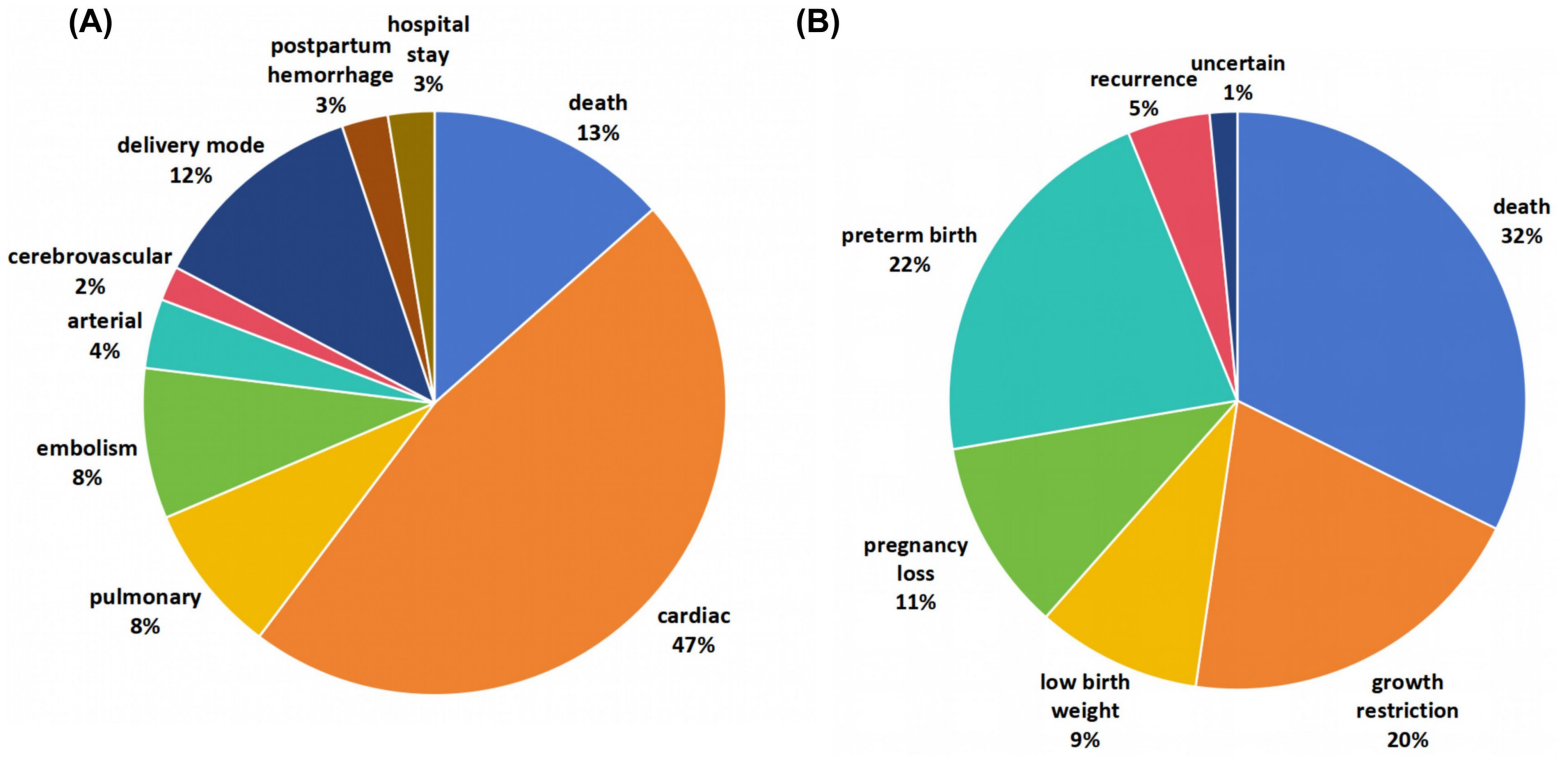
Map of adverse outcomes related to heart diseases in pregnancy. The review included 12 meta-analyses and systematic reviews, covering 156 maternal adverse outcomes and 65 offspring outcomes.

As displayed in Figure 2, the review included 12 meta-analyses and systematic reviews, covering 156 maternal adverse outcomes and 65 offspring outcomes (8, 19–29). These studies provided a broad scope of evidence on the impact of heart disease in pregnancy.

Detailed information on primary adverse outcomes is presented in Tables 1, 2, offering insights into critical maternal outcomes such as mortality, cardiac events, and cerebrovascular complications, as well as offspring’s outcomes such as stillbirth and preterm birth. Supplementary Tables 1, 2 further elaborate on secondary adverse outcomes, providing an extensive overview of less frequently reported but clinically significant complications.

**Table 1:** Heart disease in pregnancy and primary adverse outcomes of mothers.

Primary adverse outcomes of mothers	Category	Study	No. of cases/total	MA metric	Estimates	95%CI	No. of studies	Effects model	*I*^2^%
Death									
Death	Mitral stenosis (moderate)	Ducas 2020	1/287	Incidence	0.01	0.00, 0.02	6	Random	0
Death	Mitral stenosis (severe)	Ducas 2020	6/174	Incidence	0.03	0.00, 0.06	8	Random	0
Death	Aortic stenosis (severe)	Ducas 2020	2/103	Incidence	0.02	0.00, 0.05	7	Random	0
Death (in-hospital)	Cardiomyopathy vs. healthy	Eggleton 2022	138/25347	OR	126.67	43.01, 373.07	4	Random	87
Death (in-hospital)	Cardiomyopathy vs. heart disease	Eggleton 2022	150/25998	OR	4.30	3.42, 5.40	11	Fixed	0
Death (6-months)	Peripartum cardiomyopathy	Hoevelmann 2022	4,875	Prevalence	0.080	0.055, 0.108	29	Random	79.1
Death (12-months)	Peripartum cardiomyopathy	Hoevelmann 2022	4,875	Prevalence	0.098	0.006, 0.140	17	Random	80.5
Death (>1 year)	Peripartum cardiomyopathy	Koerber 2023	4,282	Prevalence (%)	9.0	6.6, 12.1	60	Random	NA
Death	Myocardial infarction	Gibson 2017	NA	Case-fatality	0.05	0.04, 0.06	9	Fixed	23.7
Death	Congenital heart disease (mild)	Hardee 2021	1/243	Morbidity (%)	0.4	0.01, 2.3	15	Random	NA
Death	Congenital heart disease (severe)	Hardee 2021	8/462	Morbidity (%)	1.7	0.8, 3.4	27	Random	NA
Maternal loss	Cardiac surgery during pregnancy	Jha 2018	13/154	Event rate (%)	11.2	6.8, 17.8	10	Random	0.0
Death	Percutaneous balloon mitral valvotomy	Sreerama 2021	11/690	Proportion	0.01	0.00, 0.76	NA	Random	NA
Cardiac									
Cardiac arrest									
Cardiac arrest	Cardiomyopathy vs. healthy	Eggleton 2022	17,528,082	OR	193.08	51.89, 718.44	3	Random	88
Cardiac arrest	Cardiomyopathy vs. heart disease	Eggleton 2022	4,873	OR	16.44	4.62, 58.54	4	Random	0
Cardiac arrest	Peripartum cardiomyopathy	Kerpen 2019	4,925	Prevalence	0.04	0.02, 0.06	6	NA	NA
Heart failure									
Heart failure	Cardiomyopathy vs. healthy	Eggleton 2022	57,439,040	OR	2804.35	2218.89, 3544.30	4	Random	95
Heart failure	Cardiomyopathy vs. heart disease	Eggleton 2022	121,653	OR	7.06	4.58, 10.88	8	Random	96
Cardiorespiratory failure or shock	Cardiomyopathy vs. healthy	Eggleton 2022	57,438,935	OR	1248.72	238.73, 6531.66	3	Random	99
Cardiorespiratory failure or shock	Cardiomyopathy vs. heart disease	Eggleton 2022	118,994	OR	19.96	6.52, 61.13	3	Random	89
Stroke (in-hospital)	Peripartum cardiomyopathy	Hoevelmann 2022	4,875	Prevalence	0.022	0.007, 0.043	NA	NA	68.5
Heart failure	Hypertrophic cardiomyopathy	Moolla 2022	43/988	Prevalence	0.05	0.03, 0.08	13	Random	67
Heart failure	Congenital heart disease (mild)	Hardee 2021	6/705	Morbidity (%)	0.9	0.3, 1.84	7	Random	NA
Heart failure	Congenital heart disease (moderate)	Hardee 2021	NA	Morbidity (%)	2.8	1.1, 7.3	12	Random	32.78
Heart failure	Congenital heart disease (severe)	Hardee 2021	NA	Morbidity (%)	15.2	7.4, 28.8	15	Random	69.26
Congestive cardiac failure	Percutaneous balloon mitral valvotomy	Sreerama 2021	1/690	Proportion	0.14	0.00, 0.08	NA	Random	NA
Surgery									
Surgical intervention	Mitral stenosis (severe)	Ducas 2020	10/174	Incidence	0.04	0.00, 0.07	8	Random	20
Percutaneous intervention	Mitral stenosis (moderate)	Ducas 2020	4/205	Incidence	0.03	0.00, 0.05	5	Random	0
Percutaneous intervention	Mitral stenosis (severe)	Ducas 2020	14/137	Incidence	0.09	0.04, 0.13	7	Random	39
Surgical intervention	Aortic stenosis (moderate)	Ducas 2020	1/81	Incidence	0.02	0.00, 0.05	4	Random	0
Percutaneous intervention	Aortic stenosis (severe)	Ducas 2020	2/103	Incidence	0.04	0.00, 0.07	7	Random	0
Heart transplant	Peripartum cardiomyopathy	Kerpen 2019	4,925	Prevalence	0.04	0.01, 0.07	19	NA	NA
LVAD implantation (>1 year)	peripartum cardiomyopathy	Koerber 2023	4,282	Prevalence (%)	7.4	2.6, 19.4	60	Random	NA
Heart transplant (>1 year)	peripartum cardiomyopathy	Koerber 2023	4,282	Prevalence (%)	10.6	4.0, 25.2	60	Random	NA
Implantable cardioverter-defibrillator (>1 year)	peripartum cardiomyopathy	Koerber 2023	4,282	Prevalence (%)	12.3	2.7, 25.2	60	Random	NA
Pulmonary									
Respiratory failure									
Respiratory failure	Cardiomyopathy vs. healthy	Eggleton 2022	57,438,935	OR	290.12	250.86, 335.53	3	random	90
Respiratory failure	Cardiomyopathy vs. heart disease	Eggleton 2022	118,994	OR	9.15	8.61, 9.72	3	fixed	45
Pulmonary oedema									
Pulmonary oedema	Mitral stenosis (moderate)	Ducas 2020	48/205	Incidence	0.18	0.02, 0.33	5	Random	92
Pulmonary oedema	Mitral stenosis (severe)	Ducas 2020	56/137	Incidence	0.37	0.23, 0.51	7	Random	63
Pulmonary oedema	Aortic stenosis (moderate)	Ducas 2020	1/19	Incidence	0.08	0.00, 0.20	3	Random	0
Pulmonary oedema	Aortic stenosis (severe)	Ducas 2020	8/69	Incidence	0.09	0.02, 0.15	6	Random	0
Pulmonary oedema	Mitral regurgitation (severe)	Ducas 2020	NA	Incidence	0.3	0.07, 0.40	2	Random	NA
Acute pulmonary edema	Percutaneous balloon mitral valvotomy	Sreerama 2021	1/690	Proportion	0.14	0.00, 0.08	NA	Random	NA
Embolism									
Pulmonary embolism	Cardiomyopathy vs. healthy	Eggleton 2022	17,527,997	OR	141.86	37.39, 538.25	2	Random	80
Pulmonary embolism	Cardiomyopathy vs. heart disease	Eggleton 2022	39,393	OR	3.19	1.73, 5.89	4	Fixed	19
Obstetrical pulmonary embolism	Cardiomyopathy vs. healthy	Eggleton 2022	57,438,935	OR	56.71	50.79, 63.32	3	Fixed	0
Obstetrical pulmonary embolism	Cardiomyopathy vs. heart disease	Eggleton 2022	118,994	OR	2.33	2.03, 2.67	3	Fixed	0
LV thrombus (in-hospital)	Peripartum cardiomyopathy	Hoevelmann 2022	4,875	Prevalence	0.090	0.065, 0.119	NA	NA	46.1
Arterial embolism (in-hospital)	Peripartum cardiomyopathy	Hoevelmann 2022	4,875	Prevalence	0.015	0.003, 0.033	NA	NA	53.9
Thromboembolism (in-hospital)	Peripartum cardiomyopathy	Hoevelmann 2022	4,875	Prevalence	0.045	0.028, 0.065	NA	NA	38.8
All-cause embolic event (in-hospital)	Peripartum cardiomyopathy	Hoevelmann 2022	4,875	Prevalence	0.061	0.038, 0.089	NA	NA	66.3
Embolism	Peripartum cardiomyopathy	Kerpen 2019	4,925	Prevalence	0.05	0.03, 0.08	12	NA	NA
Thrombolytic event	Congenital heart disease (mild)	Hardee 2021	4/372	Morbidity (%)	1.1	0.3, 2.7	4	Random	NA
Thrombolytic event	Congenital heart disease (moderate)	Hardee 2021	3/295	Morbidity (%)	1.0	0.2, 2.9	7	Random	NA
Thrombolytic event	Congenital heart disease (severe)	Hardee 2021	6/370	Morbidity (%)	1.6	0.6, 3.5	14	Random	NA
Thromboembolic events	Percutaneous balloon mitral valvotomy	Sreerama 2021	4/690	Proportion (%)	0.587	0.16, 1.48	NA	Random	NA
Cerebrovascular									
Cerebrovascular event	Cardiomyopathy vs. healthy	Eggleton 2022	57,439,040	OR	34.57	11.09, 107.77	4	Random	93
Cerebrovascular event	Cardiomyopathy vs. heart disease	Eggleton 2022	119,949	OR	1.27	0.50, 3.25	5	Random	84
Cerebrovascular accident	Peripartum cardiomyopathy	Kerpen 2019	4,925	Prevalence	0.01	0, 0.04	8	NA	NA
Postpartum hemorrhage									
Postpartum hemorrhage	Congenital heart disease (mild)	Hardee 2021	NA	Morbidity (%)	10.4	8.3, 13.0	6	Random	0.0
Postpartum hemorrhage	Congenital heart disease (moderate)	Hardee 2021	NA	Morbidity (%)	10.6	8.3, 13.5	8	Random	0.0
Postpartum hemorrhage	Congenital heart disease (severe)	Hardee 2021	39/357	Morbidity (%)	10.9	7.9, 14.6	12	Random	NA
Postpartum hemorrhage	Hypertrophic cardiomyopathy	Moolla 2022	34/925	Prevalence	0.03	0.01, 0.05	5	Random	44

MA, meta-analysis; CI, confidence interval; OR, odds ratio; RR, relative risk; MD, mean deviation; NA, not available.

**Table 2:** Heart disease in pregnancy and primary adverse outcomes of offspring.

Primary adverse outcomes of offspring	Category	Study	No. of cases/total	MA metric	Estimates	95%CI	No. of studies	Effects model	*I*^2^%
Death									
Stillbirth	Mitral stenosis (moderate)	Ducas 2020	8/287	Incidence	0.02	0.01, 0.04	6	Random	0
Stillbirth	Mitral stenosis (severe)	Ducas 2020	9/174	Incidence	0.04	0.01, 0.07	8	Random	0
Stillbirth	Aortic stenosis (severe)	Ducas 2020	1/103	Incidence	0.02	0.00, 0.05	7	Random	0
Stillbirth	Cardiomyopathy vs. Healthy	Eggleton 2023	3/676	OR	20.82	6.68, 64.95	1	Fixed	NA
Stillbirth	Cardiomyopathy vs. Cardiac disease	Eggleton 2023	11/807	OR	3.75	1.86, 7.59	3	Fixed	0
Stillbirth	Hypertrophic cardiomyopathy	Moolla 2022	14/879	Prevalence	0.01	0, 0.03	11	Random	37
Stillbirth	Percutaneous balloon mitral valvotomy	Sreerama 2021	NA	Proportion	0.0092	0.0014, 0.0214	20	Random	0
Neonatal death	Mitral stenosis (severe)	Ducas 2020	2/137	Incidence	0.02	0.00, 0.04	7	Random	0
Neonatal death	Aortic stenosis (severe)	Ducas 2020	1/103	Incidence	0.03	0.00, 0.06	7	Random	0
Neonatal death	Mitral regurgitation (severe)	Ducas 2020	1/NA	Incidence	0.06	0.01, 0.13	1	Random	NA
Neonatal death	Cardiomyopathy vs. Healthy	Eggleton 2023	12/716	OR	6.75	3.54, 12.89	2	Fixed	0
Neonatal death	Cardiomyopathy vs. Cardiac disease	Eggleton 2023	19/888	OR	2.42	1.39, 4.21	6	Fixed	0
Neonatal death	Congenital heart disease (mild)	Hardee 2021	8/773	Morbidity (%)	1.0	0.5, 2.0	12	Random	NA
Neonatal death	Congenital heart disease (moderate)	Hardee 2021	22/700	Morbidity (%)	3.1	2.0, 4.7	17	Random	NA
Neonatal death	Congenital heart disease (severe)	Hardee 2021	14/395	Morbidity (%)	3.5	2.0, 5.9	25	Random	NA
Perinatal death	Rheumatic heart disease (NYHA III/IV vs. NYHA I/II)	Liaw 2021	31/355	RR	3.23	1.92, 5.44	6	Random	0
Pregnancy loss									
Miscarriage/apontaneous abortion	Congenital heart disease (moderate)	Hardee 2021	NA	Morbidity (%)	16.1	10.6, 23.6	7	Random	63.90
Miscarriage/apontaneous abortion	Congenital heart disease (severe)	Hardee 2021	NA	Morbidity (%)	33.7	24.2, 44.7	10	Random	70.24
Therapeutic abortion	Congenital heart disease (severe)	Hardee 2021	NA	Morbidity (%)	9.5	2.2, 32.9	6	Random	86.25
Any pregnancy loss	Cardiac surgery during pregnancy	Jha 2018	49/154	Event rate (%)	33.1	25.1, 41.2	10	Random	0

MA, meta-analysis; CI, confidence interval; OR, odds ratio; RR, relative risk; NA, not available.

### Primary adverse outcomes of mothers

#### Death

The mortality incidence associated with valvular stenosis was estimated to range between 0.01 and 0.03 (8). Cardiomyopathy presented a particularly heightened risk, with an OR of 126.67 (95% CI: 43.01, 373.07) for in-hospital death compared to healthy individuals and an OR of 4.30 (95% CI: 3.42, 5.40) compared to individuals with other forms of heart disease (19). Evidence also indicated a strong association between peripartum cardiomyopathy and mortality within 6 or 12 months postpartum (23). The incidence of myocardial infarction associated with pregnancy was found to be 0.05 (95% CI: 0.04, 0.06) (21). Congenital heart disease had a mortality rate of 0.4 (95% CI: 0.01, 2.3) (22).

#### Cardiac events

The OR for cardiac arrest was notably higher in individuals with cardiomyopathy than in healthy individuals (OR: 193.08, 95% CI: 51.89, 718.44) and in those with heart disease (OR: 16.44, 95% CI: 4.62, 58.54) (19). Furthermore, cardiomyopathy was associated with a markedly elevated risk of heart failure, with an OR of 2804.35 (95% CI: 2218.89, 3544.30) compared to healthy individuals and an OR of 7.06 (95% CI: 4.58, 10.88) compared to individuals with heart disease (22). The risk of cardiorespiratory failure or shock was also significantly increased for individuals with cardiomyopathy, with an OR of 1248.72 (95% CI: 238.73, 6531.66) compared to healthy individuals and an OR of 19.96 (95% CI: 6.52, 61.13) compared to those with heart disease (19). In addition, a positive correlation was observed between the severity of congenital heart disease and the morbidity of heart failure (22). Surgical intervention for valvular stenosis was reported to have an incidence rate ranging from 0.02 to 0.09 (8).

#### Pulmonary events

The risk of respiratory failure was substantially higher in individuals with cardiomyopathy than in healthy individuals (OR: 290.12, 95% CI: 250.86, 335.53) and in those with heart disease (OR: 9.15, 95% CI: 8.61, 9.72) (19). The incidence of pulmonary edema in patients with cardiac valvular diseases ranged from 0.09 to 0.37 (8).

#### Embolism

The prevalence of embolic events in individuals with peripartum cardiomyopathy was approximately 0.05 (95% CI: 0.03, 0.08) (25). Cardiomyopathy was associated with a significantly increased risk of pulmonary embolism compared to healthy individuals (OR: 141.86, 95% CI: 37.39, 538.25) and those with heart disease (OR: 3.19, 95% CI: 1.73, 5.89) (19). Specifically, the risk of obstetrical pulmonary embolism was elevated by 55.71-fold in individuals with cardiomyopathy compared to healthy individuals and by 1.33-fold compared to those with heart disease (19). The thrombolytic morbidity among pregnant women with congenital heart disease was reported to be 1.0–1.6% (22), while it was approximately 0.587 in patients who underwent percutaneous balloon mitral valvotomy (29).

#### Cerebrovascular events

Cardiomyopathy was associated with a significantly increased risk of cerebrovascular events compared to healthy individuals (OR: 34.57, 95% CI: 11.09, 107.77). However, there was insufficient evidence to establish a significant relationship between cardiomyopathy and cerebrovascular events compared to other heart diseases (19).

#### Postpartum hemorrhage

The prevalence of postpartum hemorrhage was approximately 10% in patients with congenital heart disease (22). Among individuals with hypertrophic cardiomyopathy, the prevalence was reported to be 0.03 (95% CI: 0.01, 0.05) (28).

### Secondary adverse outcomes of mothers

#### Cardiac events

The risk of arrhythmia was 47.91 times higher in individuals with cardiomyopathy than in healthy individuals and 1.35 times higher than in those with other forms of heart disease (19). The severity of congenital heart disease appeared to be positively associated with the risk of arrhythmia (22). Cardiomyopathy was strongly associated with cardiac complications of anesthesia or sedation during labor and delivery, with an OR of 66.62 (95% CI: 53.54, 82.90) compared to healthy individuals and an OR of 7.55 (95% CI: 5.22, 10.91) compared to other forms of heart disease (19). Cardiomyopathy increased the risk of myocardial infarction substantially, with an OR of 436.34 (95% CI: 258.26, 737.21) compared to healthy individuals and an OR of 7.63 (95% CI: 6.20, 9.39) compared to other forms of heart disease (19).

#### Pulmonary events

The prevalence of respiratory support among individuals with peripartum cardiomyopathy ranged from 0.098 to 0.215 (23).

#### Arterial events

Evidence was insufficient to establish a clear association between cardiomyopathy and dissection of the aorta or another artery (19). However, hypertensive diseases of pregnancy were reported in approximately 11% of women with congenital heart disease (22), while the prevalence of pre-eclampsia/eclampsia among those with hypertrophic cardiomyopathy was 0.04 (95% CI: 0.03, 0.06) (28).

#### Delivery mode

Cardiomyopathy was significantly associated with an increased likelihood of cesarean delivery, with an OR of 2.96 (95% CI: 2.47, 3.55) compared to healthy individuals and an OR of 1.90 (95% CI: 1.62, 2.22) compared to those with other forms of heart disease (19). However, evidence was insufficient to confirm associations with elective cesarean delivery, emergency cesarean delivery, or induction of labor (19). Cardiomyopathy was associated with a 71% higher risk of instrumental delivery (OR: 1.71, 95% CI: 1.30, 2.25) and a 72% lower rate of spontaneous vaginal delivery (OR: 0.28, 95% CI: 0.23, 0.36) compared to healthy individuals (19).

#### Hospital stay

Peripartum cardiomyopathy was associated with a readmission prevalence of 0.081 (95% CI: 0.064, 0.101) at 6 months and 0.134 (95% CI: 0.082, 0.196) at 12 months postpartum (23). The mean hospital stay postdelivery was extended by 4.70 days (95% CI: 3.86, 5.53) compared to healthy individuals and by 5.18 days (95% CI: 0.70, 9.65) compared to patients with other forms of heart disease (19).

### Primary adverse outcomes of offspring

#### Death

Compared to healthy individuals, cardiomyopathy elevated the risk of stillbirth (OR: 20.82, 95% CI: 6.68, 64.95) and neonatal death (OR: 6.75, 95% CI: 3.54, 12.89) (20). Compared to other cardiac diseases, cardiomyopathy was associated with a 275% higher risk of stillbirth (OR: 3.75, 95% CI: 1.86, 7.59) and a 142% higher risk of neonatal death (OR: 2.42, 95% CI: 1.39, 4.21) (20). A severity relationship was observed between congenital heart disease risk and neonatal death (22). Rheumatic heart disease also demonstrated an association between advanced New York Heart Association (NYHA) classifications (III/IV vs. I/II) and increased perinatal death risk (RR: 3.23, 95% CI: 1.92, 5.44) (27).

#### Pregnancy loss

A severity meta-analysis highlighted a positive relationship between congenital heart disease and miscarriage/spontaneous abortion risk (22). Severe congenital heart disease was particularly associated with higher rates of miscarriage/spontaneous abortion (morbidity [%]: 33.7, 95% CI: 24.2, 44.7) and therapeutic abortion (morbidity [%]: 9.5, 95% CI: 2.2, 32.9) (22).

### Secondary adverse outcomes of offspring

#### Growth restriction

Cardiomyopathy markedly increased the risk of intrauterine growth retardation (IUGR) compared to healthy individuals (OR: 4.02, 95% CI: 2.27, 7.12) (20), and rheumatic heart disease with moderate/severe mitral stenosis was similarly associated (RR: 2.46, 95% CI: 1.02, 5.95) (27). However, no significant association was found between cardiomyopathy and other cardiac diseases (OR: 1.22, 95% CI: 0.73, 2.04) (20) or NYHA III/IV (vs. I/II) (RR: 1.53, 95% CI: 2.27, 7.13) (27). Cardiomyopathy also significantly elevated the risk of SGA births compared to healthy individuals (OR: 6.47, 95% CI: 5.32, 7.86) and patients with other cardiac diseases (OR: 2.97, 95% CI: 2.38, 3.70) (20). A severity meta-analysis showed a U-shaped relationship between congenital heart disease and SGA risk (22).

#### Low birth weight

Cardiomyopathy was significantly associated with low birth weight (<2,500 g), with increased risks compared to healthy individuals (OR: 5.37, 95% CI: 4.55, 6.33) and patients with other cardiac diseases (OR: 2.48, 95% CI: 2.02, 3.04) (20). NYHA III/IV classifications in rheumatic heart disease were also associated with a 74% increase in low birth weight risk (RR: 1.74, 95% CI: 0.98, 3.10) (20).

#### Preterm birth

Cardiomyopathy was associated with a heightened risk of preterm birth compared to healthy individuals (OR: 5.95, 95% CI: 5.01, 7.07) and patients with other forms of heart disease (OR: 2.21, 95% CI: 1.31, 3.73) (20). Congenital heart disease also showed significant positive associations with preterm birth risk, as did advanced NYHA classifications (III/IV vs. I/II) in rheumatic heart disease (RR: 2.86, 95% CI: 1.54, 5.33) (22) and moderate/severe mitral stenosis (RR: 2.05, 95% CI: 1.02, 4.11) in women with rheumatic heart disease (27).

#### Recurrence

Evidence indicated that as the severity of congenital heart disease increased, so did the risk of recurrence (22).

### Heterogeneity

Forty meta-analyses displayed very high levels of heterogeneity (*I^2^* > 75%); 55 meta-analyses presented moderate-to-high levels of heterogeneity (*I^2^* 25–75%); 51 meta-analyses demonstrated low levels of heterogeneity (*I^2^* < 25%); and 45 meta-analyses did not report heterogeneity statistics.

### Publication bias

Publication bias was assessed using Egger’s test, and no significant bias was identified in the meta-analyses included in this umbrella review.

### AMSTAR 2 and GRADE classification

Tables 3, 4 present the evaluations of the AMSTAR 2 and GRADE classification for the primary adverse outcomes affecting mothers and their offspring, while Supplementary Tables 3, 4 provide this information for secondary adverse outcomes. The overall confidence rating of the included meta-analyses and systematic reviews, as assessed by AMSTAR 2, was moderate, indicating a reasonable level of methodological quality. The quality of evidence based on the GRADE system was generally low for more than half (55.3%) of the outcomes, with the remaining outcomes categorized as having “very low”-quality evidence.

**Table 3:** Assessments of AMSTAR 2 and GRADE classification for primary adverse outcomes of mothers.

Primary adverse outcomes of mothers	Category	Study	AMASTAR 2	Grade
Death				
Death	Mitral stenosis (moderate)	Ducas 2020	Moderate	Low
Death	Mitral stenosis (severe)	Ducas 2020	Moderate	Low
Death	Aortic stenosis (severe)	Ducas 2020	Moderate	Low
Death (in-hospital)	Cardiomyopathy vs. healthy	Eggleton 2022	Moderate	Very low
Death (in-hospital)	Cardiomyopathy vs. heart disease	Eggleton 2022	High	Low
Death (6-months)	Peripartum cardiomyopathy	Hoevelmann 2022	Moderate	Very low
Death (12-months)	Peripartum cardiomyopathy	Hoevelmann 2022	Moderate	Very low
Death (>1 year)	Peripartum cardiomyopathy	Koerber 2023	Critically low	Very low
Death	Pregnancy-associated myocardial infarction	Gibson 2017	Moderate	Low
Death	Congenital heart disease (mild)	Hardee 2021	Critically low	Very low
Death	Congenital heart disease (severe)	Hardee 2021	Critically low	Very low
Maternal loss	Cardiac surgery during pregnancy	Jha 2018	Moderate	Low
Death	Percutaneous balloon mitral valvotomy	Sreerama 2021	Critically low	Very low
Cardiac				
Cardiac arrest				
Cardiac arrest	Cardiomyopathy vs. healthy	Eggleton 2022	Moderate	Very low
Cardiac arrest	Cardiomyopathy vs. heart disease	Eggleton 2022	Moderate	Low
Cardiac arrest	Peripartum cardiomyopathy	Kerpen 2019	Critically low	Very low
Heart failure				
Heart failure	Cardiomyopathy vs. healthy	Eggleton 2022	Moderate	Very low
Heart failure	Cardiomyopathy vs. heart disease	Eggleton 2022	Moderate	Very low
Cardiorespiratory failure or shock	Cardiomyopathy vs. healthy	Eggleton 2022	Moderate	Very low
Cardiorespiratory failure or shock	Cardiomyopathy vs. heart disease	Eggleton 2022	Moderate	Very low
Stroke (in-hospital)	Peripartum cardiomyopathy	Hoevelmann 2022	Low	Low
Heart failure	Hypertrophic cardiomyopathy	Moolla 2022	Low	Low
Heart failure	Congenital heart disease (mild)	Hardee 2021	Critically low	Very low
Heart failure	Congenital heart disease (moderate)	Hardee 2021	Low	Low
Heart failure	Congenital heart disease (severe)	Hardee 2021	Low	Low
Congestive cardiac failure	Percutaneous balloon mitral valvotomy	Sreerama 2021	Critically low	Very low
Surgery				
Surgical intervention	Mitral stenosis (severe)	Ducas 2020	Moderate	Low
Percutaneous intervention	Mitral stenosis (moderate)	Ducas 2020	Moderate	Low
Percutaneous intervention	Mitral stenosis (severe)	Ducas 2020	Moderate	Low
Surgical intervention	Aortic stenosis (moderate)	Ducas 2020	Critically low	Very low
Percutaneous intervention	Aortic stenosis (severe)	Ducas 2020	Critically low	Very low
Heart transplant	Peripartum cardiomyopathy	Kerpen 2019	Critically low	Very low
LVAD implantation (>1 year)	Peripartum cardiomyopathy	Koerber 2023	Critically low	Very low
Heart transplant (>1 year)	Peripartum cardiomyopathy	Koerber 2023	Critically low	Very low
Implantable cardioverter-defibrillator (>1 year)	Peripartum cardiomyopathy	Koerber 2023	Critically low	Very low
Pulmonary				
Respiratory failure				
Respiratory failure	Cardiomyopathy vs. healthy	Eggleton 2022	Moderate	Very low
Respiratory failure	Cardiomyopathy vs. heart disease	Eggleton 2022	Moderate	Low
Pulmonary oedema				
Pulmonary oedema	Mitral stenosis (moderate)	Ducas 2020	Moderate	Very low
Pulmonary oedema	Mitral stenosis (severe)	Ducas 2020	Moderate	Low
Pulmonary oedema	Aortic stenosis (moderate)	Ducas 2020	Critically low	Low
Pulmonary oedema	Aortic stenosis (severe)	Ducas 2020	Critically low	Low
Pulmonary oedema	Mitral regurgitation (severe)	Ducas 2020	Critically low	Very low
Acute pulmonary edema	Percutaneous balloon mitral valvotomy	Sreerama 2021	Critically low	Very low
Respiratory support				
Invasive ventilation (in-hospital)	Peripartum cardiomyopathy	Hoevelmann 2022	Low	Low
Inotropic support (in-hospital)	Peripartum cardiomyopathy	Hoevelmann 2022	Low	Very low
Mechanical support (in-hospital)	Peripartum cardiomyopathy	Hoevelmann 2022	Low	Low
Embolism				
Pulmonary embolism	Cardiomyopathy vs. healthy	Eggleton 2022	Moderate	Very low
Pulmonary embolism	Cardiomyopathy vs. heart disease	Eggleton 2022	Moderate	Low
Obstetrical pulmonary embolism	Cardiomyopathy vs. healthy	Eggleton 2022	Moderate	Low
Obstetrical pulmonary embolism	Cardiomyopathy vs. heart disease	Eggleton 2022	Moderate	Low
LV thrombus (in-hospital)	Peripartum cardiomyopathy	Hoevelmann 2022	Low	Low
Arterial embolism (in-hospital)	Peripartum cardiomyopathy	Hoevelmann 2022	Low	Low
Thromboembolism (in-hospital)	Peripartum cardiomyopathy	Hoevelmann 2022	Low	Low
All-cause embolic event (in-hospital)	Peripartum cardiomyopathy	Hoevelmann 2022	Low	Low
Embolism	Peripartum cardiomyopathy	Kerpen 2019	Critically low	Very low
Thrombolytic event	Congenital heart disease (mild)	Hardee 2021	Critically low	Very low
Thrombolytic event	Congenital heart disease (moderate)	Hardee 2021	Critically low	Very low
Thrombolytic event	Congenital heart disease (severe)	Hardee 2021	Critically low	Very low
Thromboembolic events	Percutaneous balloon mitral valvotomy	Sreerama 2021	Critically low	Very low
Arterial				
Dissection of aorta or another artery	Cardiomyopathy vs. healthy	Eggleton 2022	Critically low	Very low
Dissection of aorta or another artery	Cardiomyopathy vs. heart disease	Eggleton 2022	Critically low	Very low
Hypertensive disease of pregnancy	Congenital heart disease (mild)	Hardee 2021	Moderate	Low
Hypertensive disease of pregnancy	Congenital heart disease (moderate)	Hardee 2021	Low	Low
Hypertensive disease of pregnancy	Congenital heart disease (severe)	Hardee 2021	Low	Low
Pre-eclampsia/eclampsia	Hypertrophic cardiomyopathy	Moolla 2022	Moderate	Low
Cerebrovascular				
Cerebrovascular event	Cardiomyopathy vs. healthy	Eggleton 2022	Moderate	Very low
Cerebrovascular event	Cardiomyopathy vs. heart disease	Eggleton 2022	Moderate	Very low
Cerebrovascular accident	Peripartum cardiomyopathy	Kerpen 2019	Critically low	Very low
Postpartum hemorrhage				
Postpartum hemorrhage	Congenital heart disease (mild)	Hardee 2021	Moderate	Low
Postpartum hemorrhage	Congenital heart disease (moderate)	Hardee 2021	Moderate	Low
Postpartum hemorrhage	Congenital heart disease (severe)	Hardee 2021	Critically low	Very low
Postpartum hemorrhage	Hypertrophic cardiomyopathy	Moolla 2022	Low	Low

AMSTAR 2, a measurement tool to assess systematic reviews; GRADE, Grading of Recommendations Assessment, Development, and Evaluation.

**Table 4:** Assessments of AMSTAR 2 and GRADE classification for primary adverse outcomes of offspring.

Primary adverse outcomes of offspring	Category	Study	AMASTAR 2	GRADE
Death				
Stillbirth	Mitral stenosis (moderate)	Ducas 2020	Moderate	Low
Stillbirth	Mitral stenosis (severe)	Ducas 2020	Moderate	Low
Stillbirth	Aortic stenosis (severe)	Ducas 2020	Critically low	Low
Stillbirth	Cardiomyopathy vs. healthy	Eggleton 2023	Critically low	Very low
Stillbirth	Cardiomyopathy vs. cardiac disease	Eggleton 2023	Moderate	Low
Stillbirth	Hypertrophic cardiomyopathy	Moolla 2022	Low	Low
Stillbirth	Percutaneous balloon mitral valvotomy	Sreerama 2021	Moderate	Low
Neonatal death	Mitral stenosis (severe)	Ducas 2020	Critically low	Low
Neonatal death	Aortic stenosis (severe)	Ducas 2020	Critically low	Low
Neonatal death	Mitral regurgitation (severe)	Ducas 2020	Critically low	Very low
Neonatal death	Cardiomyopathy vs. healthy	Eggleton 2023	Moderate	Low
Neonatal death	Cardiomyopathy vs. cardiac disease	Eggleton 2023	Moderate	Low
Neonatal death	Congenital heart disease (mild)	Hardee 2021	Critically low	Very low
Neonatal death	Congenital heart disease (moderate)	Hardee 2021	Critically low	Very low
Neonatal death	Congenital heart disease (severe)	Hardee 2021	Critically low	Very low
Perinatal death	Rheumatic heart disease (NYHA III/IV vs. NYHA I/II)	Liaw 2021	High	Low
Pregnancy loss				
Miscarriage/apontaneous abortion	Congenital heart disease (moderate)	Hardee 2021	Low	Low
Miscarriage/apontaneous abortion	Congenital heart disease (severe)	Hardee 2021	Low	Low
Therapeutic abortion	Congenital heart disease (severe)	Hardee 2021	Low	Very low
Any pregnancy loss	Cardiac surgery during pregnancy	Jha 2018	Moderate	Low

AMSTAR 2, a measurement tool to assess systematic reviews; GRADE, Grading of Recommendations Assessment, Development, and Evaluation; NYHA, New York Heart Association.

## Discussion

### Principal findings

A total of 12 articles met the eligibility criteria, reporting 221 adverse outcomes (156 for mothers and 65 for offspring). Our review found that heart disease in pregnancy was inversely associated with maternal mortality, cardiac, pulmonary, and cerebrovascular events, as well as cesarean and instrumental deliveries, and prolonged hospital stays. On the other hand, heart disease during pregnancy was strongly associated with mortality, preterm birth, and poor intrauterine growth in offspring.

### Cardiovascular pathophysiology during pregnancy

Pregnancy induces several physiological adaptations to accommodate the growing fetus. Cardiac output can increase by up to 50%, while vascular resistance can decrease by 30%. The changes, along with a heart rate increase of approximately 10 to 20%, are crucial for supporting both maternal and fetal circulations (30). However, these adaptations may be insufficient in women with pre-existing heart disease, which can lead to complications during labor and delivery. For instance, aortocaval compression and significant blood loss during delivery may result in relative hypovolemia, reducing preload and contributing to cardiovascular instability. On the other hand, uterine contractions can cause autotransfusion, suddenly increasing preload and further stressing the cardiovascular system. The heightened stress and pain experienced during labor also exacerbate this cardiovascular strain, leading to an increased heart rate and greater demands on the heart (2). Consequently, women with underlying heart disease, particularly those with cardiomyopathy, ischemia, or cardiac arrhythmias, are at increased risk for severe cardiovascular events during pregnancy (2, 31). Notably, echocardiographic studies suggest that the rise in stroke volume and decreased afterload during pregnancy may lead to alterations in regurgitant lesions, although these changes are not always clinically significant (32).

### Comparison with existing literature

Pregnancies complicated by heart disease often fail to meet the physiological demands of pregnancy due to diminished cardiovascular reserve function. The reduced capacity to tolerate the increased cardiovascular burden may result in poor perinatal outcomes (2). Our umbrella review supports this perspective and highlights the significant risks associated with maternal heart disease. Most notably, women with cardiomyopathy face a staggering increase in mortality risk—approximately 126 times higher than the general population (19). This is primarily due to impaired systolic function, which limits the ability to increase cardiac output in response to the considerable rise in circulating blood volume required during pregnancy (33). Similarly, women with myocardial infarction face reduced cardiovascular adaptability, compounding their vulnerability (31). Meta-analyses revealed that women with mitral stenosis, congenital heart disease with severe lesions, and peripartum cardiomyopathy experience elevated mortality risks over time (8, 22, 23). In addition, the likelihood of severe cardiac events—including cardiac arrest, heart failure, cardiorespiratory failure or shock, arrhythmia, complications from anesthesia or sedation, and myocardial infarction—is markedly increased. In addition to cardiac events, risks of respiratory failure, pulmonary embolism, and cerebrovascular events are also significantly heightened in this population (19). Furthermore, pooled rates of outcomes such as surgical interventions (8, 25, 26), pulmonary edema (8, 29), respiratory support (23), thrombotic events (22, 23, 25, 29), and postpartum hemorrhage (22, 28) have been reported in several studies. However, the variability in these pooled rates complicates direct aggregation and trend assessment compared to healthy individuals. This heterogeneity underscores the challenges in evaluating risks comprehensively and highlights the need for standardized approaches in future studies to better quantify and compare outcomes. In addition, the differences in pregnancy outcomes described might be due to variations in the nature and severity of the underlying heart disease, the access to medical care, and the underlying socio-cultural environment (34). The practical management of heart disease in pregnancy should be discussed, with a focus on accurate pre-conception counseling, risk assessment, and tailored antenatal planning for women with pre-existing heart disease (35, 36). Some perspectives suggest that vaginal delivery is appropriate for the majority of women with heart disease, as it minimizes the risk of significant blood loss and avoids the complications associated with major surgery (37). The ROPAC study reported that 44% of cesarean deliveries among women with heart disease were performed for cardiac reasons (38). However, our findings revealed a higher likelihood of cesarean section among this population. This trend may reflect evolving clinical practices for managing heart disease in pregnancy across different global regions, as well as clinician-dependent decision-making, which can influence delivery methods over time.

Pregnant women with heart disease often experience impaired cardiovascular adaptation, which may compromise uteroplacental circulation and result in adverse neonatal outcomes (20). Although congenital heart disease is more prevalent in pregnancy than acquired heart disease, the associated risks are often lower due to better long-term management and relatively stable cardiovascular status (10). Conversely, acquired heart diseases, such as aortic dissection and peripartum cardiomyopathy, are associated with significantly higher rates of perinatal mortality (19, 31). Our umbrella review identified strong associations between rheumatic heart disease or cardiomyopathy in pregnancy and fatal outcomes, including stillbirth, perinatal/neonatal death, and preterm birth. Notably, the increased risk of preterm birth in these cases is frequently attributed to iatrogenic early delivery aimed at reducing maternal and fetal risks. In addition, our findings suggest an inverse relationship between rheumatic heart disease or cardiomyopathy and intrauterine growth, with affected pregnancies demonstrating reduced fetal size and birth weight (20, 27). These indicators are critical for assessing fetal growth and development, underscoring the need for enhanced monitoring in pregnancies complicated by maternal heart disease. Interestingly, some studies report a nearly linear relationship between the severity of valvular or congenital heart disease and adverse perinatal outcomes, emphasizing the importance of precise risk stratification in clinical practice (8, 22). Despite these findings, there is a notable lack of data on the long-term impact of maternal heart disease on fetal development, particularly regarding the cardiovascular and nervous systems. Addressing these gaps in future research is essential for understanding the broader implications of heart disease during pregnancy and improving outcomes for both mothers and their offspring.

### Strengths and limitations

Umbrella reviews are regarded as the most thorough evaluation of existing meta-analyses or systematic reviews, representing a top-tier level of evidence synthesis that is gaining prominence in the field of biomedical literature (39). However, several important limitations must be acknowledged. First, the majority of the evidence, as assessed by GRADE, was rated as low quality, with the remainder rated as very low or moderate quality, primarily due to the absence of RCTs. The reliance on observational studies and non-randomized designs limits the ability to draw causal inferences and weakens the overall strength of the evidence. Second, some studies exclusively report the pooled prevalence of adverse outcomes as the effect size, which complicates direct comparisons across studies. Variations in study populations, sample sizes, and measurement methods make it difficult to interpret absolute estimates objectively, thus introducing a significant source of heterogeneity. While AMSTAR 2 and GRADE showed limited correlation, the overall assessment highlighted the generally low quality of the available evidence. This underscores the need for high-quality prospective studies with standardized methodologies to provide more robust, generalizable findings.

## Conclusion and implications

Evidence shows an association between heart disease during pregnancy and adverse maternal outcomes, including death and cardiac, pulmonary, and cerebrovascular events, as well as increased mortality risk for offspring. Many meta-analyses in this field have limitations that raise concerns about their validity, highlighting the need for high-quality prospective studies.

## Data Availability

The original contributions presented in the study are included in the article/Supplementary material, further inquiries can be directed to the corresponding authors.
