# Supplementary material for: Heart disease in pregnancy and adverse outcomes: an umbrella review
Source: Front Med (Lausanne). 2025 Feb 5;12:1489991. doi: 10.3389/fmed.2025.1489991 (PMC11836018; doi:10.3389/fmed.2025.1489991)
Supplement: Supplementary file 1 [file Supplementary_file_1.docx]

**Supplementary Table 1. Heart disease in pregnancy and secondary adverse outcomes of mothers.**

| **Secondary adverse outcomes of mothers** | **Category** | **Study** | **No. of cases/total** | **MA metric** | **Estimates** | **95%CI** | **No. of studies** | **Effects model** | ***I²*%** |
| --- | --- | --- | --- | --- | --- | --- | --- | --- | --- |
| ***Cardiac*** |  |  |  |  |  |  |  |  |  |
| **arrhythmia** |  |  |  |  |  |  |  |  |  |
| new/recurrent paroxysmal arrhythmias | mitral stenosis (moderate) | Ducas 2020 | 13/205 | incidence | 0.05 | 0.01, 0.09 | 5 | random | 43 |
| new/recurrent paroxysmal arrhythmias | mitral stenosis (severe) | Ducas 2020 | 23/137 | incidence | 0.16 | 0.01, 0.25 | 7 | random | 51 |
| new/recurrent paroxysmal arrhythmias | aortic stenosis (moderate) | Ducas 2020 | 1/68 | incidence | 0.02 | 0.00, 0.05 | 2 | random | 0 |
| new/recurrent paroxysmal arrhythmias | aortic stenosis (severe) | Ducas 2020 | 3/87 | incidence | 0.04 | 0.00, 0.07 | 5 | random | 0 |
| arrhythmia | cardiomyopathy vs healthy | Eggleton 2022 | 57439040 | OR | 48.91 | 44.60, 53.64 | 4 | fixed | 67 |
| arrhythmia | cardiomyopathy vs heart disease | Eggleton 2022 | 121257 | OR | 2.35 | 1.99, 2.78 | 6 | random | 78 |
| sustained ventricular tachycardia | hypertrophic cardiomyopathy | Moolla 2022 | 5/410 | prevalence | 0.01 | 0, 0.01 | 7 | random | 0 |
| ventricular tachycardia (including NSVT and sustained VT) | hypertrophic cardiomyopathy | Moolla 2022 | 54/862 | prevalence | 0.06 | 0.04, 0.08 | 9 | random | 25 |
| atrial fibrillation | hypertrophic cardiomyopathy | Moolla 2022 | 22/411 | prevalence | 0.04 | 0.02, 0.06 | 6 | random | 19 |
| arrhythmia | congenital heart disease (mild) | Hardee 2021 | NA | morbidity (%) | 3.2 | 2.0, 5.0 | 7 | random | 0 |
| arrhythmia | congenital heart disease (moderate) | Hardee 2021 | NA | morbidity (%) | 8.1 | 4.1, 15.3 | 12 | random | 61.56 |
| arrhythmia | congenital heart disease (severe) | Hardee 2021 | NA | morbidity (%) | 12.6 | 7.4, 20.7 | 17 | random | 58.44 |
| arrhythmia | peripartum cardiomyopathy | Kerpen 2019 | 4925 | prevalence | 0.08 | 0.04, 0.15 | 15 | NA | NA |
| arrhythmia | percutaneous balloon mitral valvotomy | Sreerama 2021 | 2/690 | proportion | 0.29 | 0.04, 1.04 | NA | random | NA |
| atrial shunt | percutaneous balloon mitral valvotomy | Sreerama 2021 | 9/690 | proportion | 0.03 | 0.00, 0.57 | NA | random | NA |
| **anesthesia or sedation** |  |  |  |  |  |  |  |  |  |
| cardiac complications of anesthesia or sedation during labor and delivery | cardiomyopathy vs healthy | Eggleton 2022 | 42191807 | OR | 66.62 | 53.54, 82.90 | 2 | fixed | 0 |
| cardiac complications of anesthesia or sedation during labor and delivery | cardiomyopathy vs heart disease | Eggleton 2022 | 85166 | OR | 7.55 | 5.22, 10.91 | 2 | fixed | 0 |
| **endocarditis** |  |  |  |  |  |  |  |  |  |
| endocarditis | congenital heart disease (mild) | Hardee 2021 | 2/700 | morbidity (%) | 0.3 | 0.0, 1.03 | 6 | random | NA |
| endocarditis | congenital heart disease (moderate) | Hardee 2021 | 1/520 | morbidity (%) | 0.2 | 0.0, 1.07 | 7 | random | NA |
| endocarditis | congenital heart disease (severe) | Hardee 2021 | 1/172 | morbidity (%) | 0.6 | 0.00, 3.20 | 10 | random | NA |
| **mitral regurgitation** |  |  |  |  |  |  |  |  |  |
| mitral regurgitation | percutaneous balloon mitral valvotomy | Sreerama 2021 | NA | proportion | 0.127 | 0.073, 0.191 | 19 | random | 77 |
| **myocardial infarction** |  |  |  |  |  |  |  |  |  |
| myocardial infarction | cardiomyopathy vs healthy | Eggleton 2022 | 57439040 | OR | 436.34 | 258.26, 737.21 | 4 | random | 87 |
| myocardial infarction | cardiomyopathy vs heart disease | Eggleton 2022 | 119949 | OR | 7.63 | 6.20, 9.39 | 5 | fixed | 0 |
| **NYHA III-IV** |  |  |  |  |  |  |  |  |  |
| NYHA III-IV | mitral stenosis (moderate) | Ducas 2020 | 44/158 | incidence | 0.28 | 0.00, 0.56 | 3 | random | 96 |
| NYHA III-IV | mitral stenosis (severe) | Ducas 2020 | 87/121 | incidence | 0.78 | 0.54, 1.00 | 5 | random | 93 |
| NYHA III-IV | aortic stenosis (moderate) | Ducas 2020 | 6/74 | incidence | 0.08 | 0.02, 0.15 | 3 | random | 0 |
| NYHA III-IV | aortic stenosis (severe) | Ducas 2020 | 13/84 | incidence | 0.22 | 0.05, 0.40 | 5 | random | 83 |
| **restenosis** |  |  |  |  |  |  |  |  |  |
| restenosis | percutaneous balloon mitral valvotomy | Sreerama 2021 | NA | proportion | 0.024 | 0, 0.007 | 12 | random | 63 |
| **syncope** |  |  |  |  |  |  |  |  |  |
| syncope | hypertrophic cardiomyopathy | Moolla 2022 | 26/375 | prevalence | 0.09 | 0.03, 0.14 | 5 | random | 45 |
| **others** |  |  |  |  |  |  |  |  |  |
| combined major adverse cardiovascular events | cardiomyopathy vs healthy | Eggleton 2022 | 11587/25347 | OR | 206.64 | 192.09, 222.28 | 4 | random | 73 |
| combined major adverse cardiovascular events | cardiomyopathy vs heart disease | Eggleton 2022 | 11595/25412 | OR | 7.09 | 6.08, 8.27 | 5 | random | 88 |
| maternal complications | cardiac surgery during pregnancy | Jha 2018 | 20/138 | event rate (%) | 8.8 | 2.8, 24.2 | 8 | random | 66.6 |
| major adverse cardiac events (>1 year) | peripartum cardiomyopathy | Koerber 2023 | 4282 | prevalence (%) | 3.0 | 1.2, 7.3 | 60 | random | NA |
| left ventricular ejection fraction | cardiomyopathy vs healthy | Eggleton 2022 | 119 | MD | -25.17 | -38.98, -11.35 | 2 | random | 96 |
| left ventricular ejection fraction | cardiomyopathy vs heart disease | Eggleton 2022 | 75 | MD | -14.15 | -16.70, -11.59 | 2 | fixed | 72 |
| change in LVEF from baseline (6-months) | peripartum cardiomyopathy | Hoevelmann 2022 | 4875 | prevalence | 0.177 | 0.157, 0.196 | NA | NA | 85.0 |
| recovered LVEF (≥50%) (6-months) | peripartum cardiomyopathy | Hoevelmann 2022 | 4875 | prevalence | 0.444 | 0.362, 0.528 | 29 | random | 91.4 |
| change in LVEF from baseline (12-months) | peripartum cardiomyopathy | Hoevelmann 2022 | 4875 | prevalence | 0.187 | 0.157, 0.216 | NA | NA | 73.2 |
| recovered LVEF (≥50%) (12-months) | peripartum cardiomyopathy | Hoevelmann 2022 | 4875 | prevalence | 0.587 | 0.481, 0.689 | 10 | random | 75.8 |
| myocardial recovery (>1 year) | peripartum cardiomyopathy | Koerber 2023 | 4282 | prevalence (%) | 47.1 | 38.5, 55.9 | 60 | random | NA |
| ***Pulmonary*** |  |  |  |  |  |  |  |  |  |
| **respiratory support** |  |  |  |  |  |  |  |  |  |
| invasive ventilation (in-hospital) | peripartum cardiomyopathy | Hoevelmann 2022 | 4875 | prevalence | 0.098 | 0.030, 0.192 | NA | NA | 67.3 |
| inotropic support (in-hospital) | peripartum cardiomyopathy | Hoevelmann 2022 | 4875 | prevalence | 0.215 | 0.079, 0.390 | NA | NA | 91.1 |
| mechanical support (in-hospital) | peripartum cardiomyopathy | Hoevelmann 2022 | 4875 | prevalence | 0.031 | 0.020, 0.044 | NA | NA | 10.3 |
| ***Arterial*** |  |  |  |  |  |  |  |  |  |
| dissection of aorta or another artery | cardiomyopathy vs healthy | Eggleton 2022 | 2956 | OR | 0.13 | 0.01, 2.27 | 1 | random | NA |
| dissection of aorta or another artery | cardiomyopathy vs heart disease | Eggleton 2022 | 3871 | OR | 0.11 | 0.01, 1.90 | 1 | random | NA |
| hypertensive disease of pregnancy | congenital heart disease (mild) | Hardee 2021 | NA | morbidity (%) | 11.3 | 9.2, 14.0 | 4 | random | 0.0 |
| hypertensive disease of pregnancy | congenital heart disease (moderate) | Hardee 2021 | NA | morbidity (%) | 11.8 | 8.9, 15.5 | 7 | random | 19.79 |
| hypertensive disease of pregnancy | congenital heart disease (severe) | Hardee 2021 | NA | morbidity (%) | 10.3 | 5.2, 19.4 | 12 | random | 56.13 |
| pre-eclampsia/eclampsia | hypertrophic cardiomyopathy | Moolla 2022 | 42/925 | prevalence | 0.04 | 0.03, 0.06 | 5 | random | 0 |
| ***Delivery mode*** |  |  |  |  |  |  |  |  |  |
| cesarean delivery | cardiomyopathy vs healthy | Eggleton 2022 | 57439222 | OR | 2.96 | 2.47, 3.55 | 5 | random | 95 |
| cesarean delivery | cardiomyopathy vs heart disease | Eggleton 2022 | 121268 | OR | 1.90 | 1.62, 2.22 | 6 | random | 91 |
| cesarean section | hypertrophic cardiomyopathy | Moolla 2022 | 544/1109 | prevalence | 0.43 | 0.35, 0.52 | 13 | random | 84 |
| cesarean section | percutaneous balloon mitral valvotomy | Sreerama 2021 | NA | proportion | 0.121 | 0.036, 0.238 | NA | random | NA |
| elective cesarean delivery | cardiomyopathy vs heart disease | Eggleton 2022 | 753 | OR | 1.05 | 0.63, 1.74 | 1 | fixed | 0 |
| cesarean section for cardiovascular indications | hypertrophic cardiomyopathy | Moolla 2022 | 11/74 | prevalence | 0.14 | 0.03, 0.25 | 6 | random | 35 |
| emergency cesarean delivery | cardiomyopathy vs healthy | Eggleton 2022 | 105 | OR | 1.49 | 0.60, 3.71 | 1 | random | NA |
| emergency cesarean delivery | cardiomyopathy vs heart disease | Eggleton 2022 | 955 | OR | 1.33 | 0.22, 8.10 | 2 | random | 88 |
| cesarean section | congenital heart disease (mild) | Hardee 2021 | NA | morbidity (%) | 22.5 | 12.3, 37.7 | 5 | random | 84.50 |
| cesarean section | congenital heart disease (moderate) | Hardee 2021 | NA | morbidity (%) | 30.8 | 19.3, 45.3 | 9 | random | 59.84 |
| cesarean section | congenital heart disease (severe) | Hardee 2021 | NA | morbidity (%) | 52.0 | 35.8, 67.8 | 12 | random | 70.19 |
| cesarean delivery | cardiac surgery during pregnancy | Jha 2018 | 25/75 | event rate (%) | 33.8 | 19.1, 52.4 | 6 | random | 49 |
| instrumental delivery | cardiomyopathy vs healthy | Eggleton 2022 | 2280954 | OR | 1.71 | 1.30, 2.25 | 2 | fixed | 41 |
| instrumental delivery | cardiomyopathy vs heart disease | Eggleton 2022 | 4359 | OR | 0.81 | 0.61, 1.07 | 4 | fixed | 31 |
| spontaneous vaginal delivery | cardiomyopathy vs healthy | Eggleton 2022 | 17528082 | OR | 0.28 | 0.23, 0.36 | 3 | random | 77 |
| spontaneous vaginal delivery | cardiomyopathy vs heart disease | Eggleton 2022 | 34976 | OR | 0.61 | 0.37, 1.01 | 4 | fixed | 72 |
| induction of labor | cardiomyopathy vs healthy | Eggleton 2022 | 105 | OR | 1.46 | 0.63, 3.40 | 1 | random | NA |
| induction of labor | cardiomyopathy vs heart disease | Eggleton 2022 | 102 | OR | 0.91 | 0.40, 2.09 | 1 | random | NA |
| ***Hospital stay*** |  |  |  |  |  |  |  |  |  |
| all-cause readmission (6-months) | peripartum cardiomyopathy | Hoevelmann 2022 | 4875 | prevalence | 0.081 | 0.064, 0.101 | NA | NA | 0.0 |
| all-cause readmission (12-months) | peripartum cardiomyopathy | Hoevelmann 2022 | 4875 | prevalence | 0.134 | 0.082, 0.196 | NA | NA | 73.1 |
| mean length of hospital stay postdelivery | cardiomyopathy vs healthy | Eggleton 2022 | 713 | MD | 4.70 | 3.86, 5.53 | 2 | fixed | 0 |
| mean length of hospital stay postdelivery | cardiomyopathy vs heart disease | Eggleton 2022 | 713 | MD | 5.18 | 0.70, 9.65 | 2 | random | 77 |

Note: MA, meta-analysis; CI, confidence interval; OR, odds ratio; RR, relative risk; MD, mean deviation; NA, not available; NYHA, New York Heart Association; LVEF, left ventricular ejection fraction; LVAD, left ventricular assist device.

**Supplementary Table 2. Heart disease in pregnancy and secondary adverse outcomes of offspring.**

| **Primary adverse outcomes of offspring** | **Category** | **Study** | **No. of cases/total** | **MA metric** | **Estimates** | **95%CI** | **No. of studies** | **Effects model** | ***I²*%** |
| --- | --- | --- | --- | --- | --- | --- | --- | --- | --- |
| ***Growth restriction*** |  |  |  |  |  |  |  |  |  |
| IUGR | cardiomyopathy vs healthy | Eggleton 2023 | 2280849 | OR | 4.02 | 2.27, 7.12 | 1 | NA | NA |
| IUGR | cardiomyopathy vs cardiac disease | Eggleton 2023 | 4791 | OR | 1.22 | 0.73, 2.04 | 3 | fixed | 67 |
| IUGR | rheumatic heart disease (NYHA III/IV vs NYHA I/II) | Liaw 2021 | 67/249 | RR | 1.53 | 0.84, 2.80 | 3 | random | 46 |
| IUGR | rheumatic heart disease (moderate/severe mitral stenosis vs mild mitral stenosis) | Liaw 2021 | 27/215 | RR | 2.46 | 1.02, 5.95 | 3 | random | 0 |
| SGA | mitral stenosis (moderate) | Ducas 2020 | 19/154 | incidence | 0.13 | 0.05, 0.21 | 3 | random | 35 |
| SGA | mitral stenosis (severe) | Ducas 2020 | 16/94 | incidence | 0.14 | 0.05, 0.23 | 5 | random | 35 |
| SGA | aortic stenosis (moderate) | Ducas 2020 | 4/75 | incidence | 0.05 | 0.00, 0.13 | 3 | random | 20 |
| SGA | aortic stenosis (severe) | Ducas 2020 | 9/97 | incidence | 0.07 | 0.01, 0.14 | 6 | random | 42 |
| SGA | cardiomyopathy vs healthy | Eggleton 2023 | 2280957 | OR | 6.47 | 5.32, 7.86 | 2 | fixed | 40 |
| SGA | cardiomyopathy vs cardiac disease | Eggleton 2023 | 4292 | OR | 2.97 | 2.38, 3.70 | 4 | fixed | 47 |
| SGA | congenital heart disease (mild) | Hardee 2021 | NA | morbidity (%) | 14.6 | 11.0, 19.2 | 4 | random | 55.74 |
| SGA | congenital heart disease (moderate) | Hardee 2021 | NA | morbidity (%) | 13.8 | 9.3, 20.1 | 9 | random | 61.42 |
| SGA | congenital heart disease (severe) | Hardee 2021 | NA | morbidity (%) | 35.8 | 24.0, 49.6 | 14 | random | 69.5 |
| ***Low birth weight*** |  |  |  |  |  |  |  |  |  |
| birth weight | cardiomyopathy vs healthy | Eggleton 2023 | 2529 | MD | -478.19 | -608.10, -348.28 | 2 | fixed | 0 |
| birth weight | cardiomyopathy vs cardiac disease | Eggleton 2023 | 576 | MD | -386.64 | -712.56, -60.72 | 5 | random | 81 |
| birth weight (<2500g) | cardiomyopathy vs healthy | Eggleton 2023 | 2283270 | OR | 5.37 | 4.55, 6.33 | 2 | fixed | 0 |
| birth weight (<2500g) | cardiomyopathy vs cardiac disease | Eggleton 2023 | 4183 | OR | 2.48 | 2.02, 3.04 | 3 | fixed | 28 |
| low birth weight | rheumatic heart disease (NYHA III/IV vs NYHA I/II) | Liaw 2021 | 70/151 | RR | 1.74 | 0.98, 3.10 | 4 | random | 85 |
| low birth weight | percutaneous balloon mitral valvotomy | Sreerama 2021 | NA | proportion | 0.0539 | 0.0018, 0.1472 | 19 | random | 91 |
| ***Preterm birth*** |  |  |  |  |  |  |  |  |  |
| preterm birth | mitral stenosis (moderate) | Ducas 2020 | 23/205 | incidence | 0.10 | 0.02, 0.17 | 5 | random | 70 |
| preterm birth | mitral stenosis (severe) | Ducas 2020 | 24/137 | incidence | 0.18 | 0.07, 0.29 | 7 | random | 69 |
| preterm birth | aortic stenosis (moderate) | Ducas 2020 | 11/81 | incidence | 0.13 | 0.06, 0.20 | 4 | random | 0 |
| preterm birth | aortic stenosis (severe) | Ducas 2020 | 17/103 | incidence | 0.14 | 0.04, 0.24 | 7 | random | 83 |
| preterm birth | cardiomyopathy vs healthy | Eggleton 2023 | 184/713 | OR | 5.95 | 5.01, 7.07 | 2 | fixed | 32 |
| preterm birth | cardiomyopathy vs heart disease | Eggleton 2023 | 253/965 | OR | 2.21 | 1.31, 3.73 | 9 | random | 77 |
| preterm birth | congenital heart disease (mild) | Hardee 2021 | NA | morbidity (%) | 5.8 | 4.3, 7.9 | 4 | random | 0 |
| preterm birth | congenital heart disease (moderate) | Hardee 2021 | NA | morbidity (%) | 13.9 | 11.4, 17.0 | 9 | random | 0 |
| preterm birth | congenital heart disease (severe) | Hardee 2021 | NA | morbidity (%) | 50.5 | 36.4, 64.6 | 17 | random | 76.72 |
| preterm birth | cardiac surgery during pregnancy | Jha 2018 | 37/123 | event rate (%) | 28 | 15.6, 45 | 8 | random | 57.7 |
| preterm birth | rheumatic heart disease (NYHA III/IV vs NYHA I/II) | Liaw 2021 | 64/204 | RR | 2.86 | 1.54, 5.33 | 5 | random | 64 |
| preterm birth | rheumatic heart disease (moderate/severe mitral stenosis vs mild mitral stenosis) | Liaw 2021 | 32/215 | RR | 2.05 | 1.02, 4.11 | 3 | random | 0 |
| preterm birth | hypertrophic cardiomyopathy | Moolla 2022 | 130/595 | prevalence | 0.22 | 0.18, 0.25 | 7 | random | 0 |
| preterm birth | percutaneous balloon mitral valvotomy | Sreerama 2021 | NA | proportion | 0.0388 | 0.0058, 0.0897 | 20 | random | 82 |
| ***Recurrence*** |  |  |  |  |  |  |  |  |  |
| recurrence | congenital heart disease (mild) | Hardee 2021 | 8/364 | morbidity (%) | 2.2 | 1.0, 4.3 | 4 | random | NA |
| recurrence | congenital heart disease (moderate) | Hardee 2021 | 16/390 | morbidity (%) | 4.1 | 2.4, 6.6 | 9 | random | NA |
| recurrence | congenital heart disease (severe) | Hardee 2021 | 9/297 | morbidity (%) | 4.7 | 2.6, 7.9 | 15 | random | NA |
| ***Uncertain*** |  |  |  |  |  |  |  |  |  |
| neonatal complications | cardiac surgery during pregnancy | Jha 2018 | 18/154 | event rate (%) | 10.8 | 4.2, 25.2 | 10 | random | 63.9 |

Note: MA, meta-analysis; CI, confidence interval; OR, odds ratio; RR, relative risk; NA, not available; IUGR, intrauterine growth retardation; SGA, small for gestational age; NYHA, New York Heart Association.

**Supplementary Table 3. Assessments of AMSTAR 2 and GRADE classification for secondary adverse outcomes of mothers.**

| **Secondary adverse outcomes of mothers** | **Category** | **Study** | **AMASTAR 2** | **GRADE** |
| --- | --- | --- | --- | --- |
| ***Cardiac*** |  |  |  |  |
| **arrhythmia** |  |  |  |  |
| new/recurrent paroxysmal arrhythmias | mitral stenosis (moderate) | Ducas 2020 | Moderate | Low |
| new/recurrent paroxysmal arrhythmias | mitral stenosis (severe) | Ducas 2020 | Moderate | Low |
| new/recurrent paroxysmal arrhythmias | aortic stenosis (moderate) | Ducas 2020 | Critically low | Very low |
| new/recurrent paroxysmal arrhythmias | aortic stenosis (severe) | Ducas 2020 | Critically low | Low |
| arrhythmia | cardiomyopathy vs healthy | Eggleton 2022 | Moderate | Low |
| arrhythmia | cardiomyopathy vs heart disease | Eggleton 2022 | Moderate | Low |
| sustained ventricular tachycardia | hypertrophic cardiomyopathy | Moolla 2022 | Moderate | Low |
| ventricular tachycardia (including NSVT and sustained VT) | hypertrophic cardiomyopathy | Moolla 2022 | Low | Low |
| atrial fibrillation | hypertrophic cardiomyopathy | Moolla 2022 | Low | Low |
| arrhythmia | congenital heart disease (mild) | Hardee 2021 | Moderate | Low |
| arrhythmia | congenital heart disease (moderate) | Hardee 2021 | Low | Low |
| arrhythmia | congenital heart disease (severe) | Hardee 2021 | Low | Low |
| arrhythmia | peripartum cardiomyopathy | Kerpen 2019 | Critically low | Very low |
| arrhythmia | percutaneous balloon mitral valvotomy | Sreerama 2021 | Critically low | Very low |
| atrial shunt | percutaneous balloon mitral valvotomy | Sreerama 2021 | Critically low | Very low |
| **anesthesia or sedation** |  |  |  |  |
| cardiac complications of anesthesia or sedation during labor and delivery | cardiomyopathy vs healthy | Eggleton 2022 | Moderate | Low |
| cardiac complications of anesthesia or sedation during labor and delivery | cardiomyopathy vs heart disease | Eggleton 2022 | Moderate | Low |
| **endocarditis** |  |  |  |  |
| endocarditis | congenital heart disease (mild) | Hardee 2021 | Critically low | Very low |
| endocarditis | congenital heart disease (moderate) | Hardee 2021 | Critically low | Very low |
| endocarditis | congenital heart disease (severe) | Hardee 2021 | Critically low | Very low |
| **mitral regurgitation** |  |  |  |  |
| mitral regurgitation | percutaneous balloon mitral valvotomy | Sreerama 2021 | Low | Very low |
| **myocardial infarction** |  |  |  |  |
| myocardial infarction | cardiomyopathy vs healthy | Eggleton 2022 | Moderate | Very low |
| myocardial infarction | cardiomyopathy vs heart disease | Eggleton 2022 | Moderate | Low |
| **NYHA III-IV** |  |  |  |  |
| NYHA III-IV | mitral stenosis (moderate) | Ducas 2020 | Critically low | Very low |
| NYHA III-IV | mitral stenosis (severe) | Ducas 2020 | Critically low | Very low |
| NYHA III-IV | aortic stenosis (moderate) | Ducas 2020 | Critically low | Low |
| NYHA III-IV | aortic stenosis (severe) | Ducas 2020 | Critically low | Very low |
| **restenosis** |  |  |  |  |
| restenosis | percutaneous balloon mitral valvotomy | Sreerama 2021 | Low | Low |
| **syncope** |  |  |  |  |
| syncope | hypertrophic cardiomyopathy | Moolla 2022 | Low | Low |
| **others** |  |  |  |  |
| combined major adverse cardiovascular events | cardiomyopathy vs healthy | Eggleton 2022 | Moderate | Low |
| combined major adverse cardiovascular events | cardiomyopathy vs heart disease | Eggleton 2022 | Moderate | Very low |
| maternal complications | cardiac surgery during pregnancy | Jha 2018 | Low | Low |
| major adverse cardiac events (>1 year) | peripartum cardiomyopathy | Koerber 2023 | Critically low | Very low |
| left ventricular ejection fraction | cardiomyopathy vs healthy | Eggleton 2022 | Moderate | Very low |
| left ventricular ejection fraction | cardiomyopathy vs heart disease | Eggleton 2022 | Moderate | Low |
| change in LVEF from baseline (6-months) | peripartum cardiomyopathy | Hoevelmann 2022 | Low | Very low |
| recovered LVEF (≥50%) (6-months) | peripartum cardiomyopathy | Hoevelmann 2022 | Moderate | Very low |
| change in LVEF from baseline (12-months) | peripartum cardiomyopathy | Hoevelmann 2022 | Low | Low |
| recovered LVEF (≥50%) (12-months) | peripartum cardiomyopathy | Hoevelmann 2022 | Moderate | Very low |
| myocardial recovery (>1 year) | peripartum cardiomyopathy | Koerber 2023 | Critically low | Very low |
| ***Delivery mode*** |  |  |  |  |
| cesarean delivery | cardiomyopathy vs healthy | Eggleton 2022 | Moderate | Very low |
| cesarean delivery | cardiomyopathy vs heart disease | Eggleton 2022 | Moderate | Very low |
| cesarean section | hypertrophic cardiomyopathy | Moolla 2022 | Low | Very low |
| cesarean section | percutaneous balloon mitral valvotomy | Sreerama 2021 | Critically low | Very low |
| elective cesarean delivery | cardiomyopathy vs heart disease | Eggleton 2022 | Critically low | Very low |
| cesarean section for cardiovascular indications | hypertrophic cardiomyopathy | Moolla 2022 | Low | Low |
| emergency cesarean delivery | cardiomyopathy vs healthy | Eggleton 2022 | Critically low | Very low |
| emergency cesarean delivery | cardiomyopathy vs heart disease | Eggleton 2022 | Moderate | Very low |
| cesarean section | congenital heart disease (mild) | Hardee 2021 | Critically low | Very low |
| cesarean section | congenital heart disease (moderate) | Hardee 2021 | Critically low | Low |
| cesarean section | congenital heart disease (severe) | Hardee 2021 | Critically low | Low |
| cesarean delivery | cardiac surgery during pregnancy | Jha 2018 | Low | Low |
| instrumental delivery | cardiomyopathy vs healthy | Eggleton 2022 | Moderate | Low |
| instrumental delivery | cardiomyopathy vs heart disease | Eggleton 2022 | Moderate | Low |
| spontaneous vaginal delivery | cardiomyopathy vs healthy | Eggleton 2022 | Moderate | Very low |
| spontaneous vaginal delivery | cardiomyopathy vs heart disease | Eggleton 2022 | Moderate | Low |
| induction of labor | cardiomyopathy vs healthy | Eggleton 2022 | Critically low | Very low |
| induction of labor | cardiomyopathy vs heart disease | Eggleton 2022 | Critically low | Very low |
| ***Hospital stay*** |  |  |  |  |
| all-cause readmission (6-months) | peripartum cardiomyopathy | Hoevelmann 2022 | Low | Low |
| all-cause readmission (12-months) | peripartum cardiomyopathy | Hoevelmann 2022 | Low | Low |
| mean length of hospital stay postdelivery | cardiomyopathy vs healthy | Eggleton 2022 | Moderate | Low |
| mean length of hospital stay postdelivery | cardiomyopathy vs heart disease | Eggleton 2022 | Moderate | Very low |

Note: AMSTAR 2, a measurement tool to assess systematic reviews; GRADE, Grading of Recommendations Assessment, Development, and Evaluation; NYHA, New York Heart Association; LVEF, left ventricular ejection fraction; LVAD, left ventricular assist device.

**Supplementary Table 4. Assessments of AMSTAR 2 and GRADE classification for secondary adverse outcomes of offspring.**

| **Secondary adverse outcomes of offspring** | **Category** | **Study** | **AMASTAR 2** | **GRADE** |
| --- | --- | --- | --- | --- |
| ***Growth restriction*** |  |  |  |  |
| IUGR | cardiomyopathy vs healthy | Eggleton 2023 | Critically low | Very low |
| IUGR | cardiomyopathy vs cardiac disease | Eggleton 2023 | Moderate | Low |
| IUGR | rheumatic heart disease (NYHA III/IV vs NYHA I/II) | Liaw 2021 | Moderate | Low |
| IUGR | rheumatic heart disease (moderate/severe mitral stenosis vs mild mitral stenosis) | Liaw 2021 | High | Low |
| SGA | mitral stenosis (moderate) | Ducas 2020 | Moderate | Low |
| SGA | mitral stenosis (severe) | Ducas 2020 | Critically low | Low |
| SGA | aortic stenosis (moderate) | Ducas 2020 | Critically low | Low |
| SGA | aortic stenosis (severe) | Ducas 2020 | Critically low | Low |
| SGA | cardiomyopathy vs healthy | Eggleton 2023 | Moderate | Low |
| SGA | cardiomyopathy vs cardiac disease | Eggleton 2023 | Moderate | Low |
| SGA | congenital heart disease (mild) | Hardee 2021 | Low | Low |
| SGA | congenital heart disease (moderate) | Hardee 2021 | Low | Low |
| SGA | congenital heart disease (severe) | Hardee 2021 | Low | Low |
| ***Low birth weight*** |  |  |  |  |
| birth weight | cardiomyopathy vs healthy | Eggleton 2023 | Moderate | Low |
| birth weight | cardiomyopathy vs cardiac disease | Eggleton 2023 | Moderate | Very low |
| birth weight (<2500g) | cardiomyopathy vs healthy | Eggleton 2023 | Moderate | Low |
| birth weight (<2500g) | cardiomyopathy vs cardiac disease | Eggleton 2023 | Moderate | Low |
| low birth weight | rheumatic heart disease (NYHA III/IV vs NYHA I/II) | Liaw 2021 | Moderate | Very low |
| low birth weight | percutaneous balloon mitral valvotomy | Sreerama 2021 | Low | Very low |
| ***Preterm birth*** |  |  |  |  |
| preterm birth | mitral stenosis (moderate) | Ducas 2020 | Critically low | Low |
| preterm birth | mitral stenosis (severe) | Ducas 2020 | Critically low | Low |
| preterm birth | aortic stenosis (moderate) | Ducas 2020 | Critically low | Low |
| preterm birth | aortic stenosis (severe) | Ducas 2020 | Critically low | Very low |
| preterm birth | cardiomyopathy vs healthy | Eggleton 2023 | Moderate | Low |
| preterm birth | cardiomyopathy vs heart disease | Eggleton 2023 | Moderate | Very low |
| preterm birth | congenital heart disease (mild) | Hardee 2021 | Moderate | Low |
| preterm birth | congenital heart disease (moderate) | Hardee 2021 | Moderate | Low |
| preterm birth | congenital heart disease (severe) | Hardee 2021 | Low | Very low |
| preterm birth | cardiac surgery during pregnancy | Jha 2018 | Low | Low |
| preterm birth | rheumatic heart disease (NYHA III/IV vs NYHA I/II) | Liaw 2021 | Moderate | Low |
| preterm birth | rheumatic heart disease (moderate/severe mitral stenosis vs mild mitral stenosis) | Liaw 2021 | High | Low |
| preterm birth | hypertrophic cardiomyopathy | Moolla 2022 | Moderate | Low |
| preterm birth | percutaneous balloon mitral valvotomy | Sreerama 2021 | Low | Very low |
| ***Recurrence*** |  |  |  |  |
| recurrence | congenital heart disease (mild) | Hardee 2021 | Critically low | Very low |
| recurrence | congenital heart disease (moderate) | Hardee 2021 | Critically low | Very low |
| recurrence | congenital heart disease (severe) | Hardee 2021 | Critically low | Very low |
| ***Uncertain*** |  |  |  |  |
| neonatal complications | cardiac surgery during pregnancy | Jha 2018 | Low | Low |

Note: AMSTAR 2, a measurement tool to assess systematic reviews; GRADE, Grading of Recommendations Assessment, Development, and Evaluation; IUGR, intrauterine growth retardation; SGA, small for gestational age; NYHA, New York Heart Association.
